# Behavioral Adaptation to Improved Environmental Quality: Evidence From a Sanitation Intervention

**DOI:** 10.1002/hec.70016

**Published:** 2025-07-19

**Authors:** Lisa Cameron, An Huang, Paulo Santos, Milan Thomas

**Affiliations:** ^1^ Melbourne Institute of Applied Economic and Social Research University of Melbourne Parkville Australia; ^2^ School of Economics Sichuan University Chengdu China; ^3^ Department of Economics Monash Business School, Monash University Caulfield East Australia; ^4^ Department of Economics Monash University Clayton Australia; ^5^ Economic Research and Development Impact Department Asian Development Bank Mandaluyong Philippines

**Keywords:** firewood collection, height‐for‐age, WASH, water boiling

## Abstract

This paper investigates behavioral adaptation to local improvements in environmental quality. Using exogenous variation in village sanitation coverage generated by the randomised allocation of financial incentives to latrine construction in Lao PDR, we find that the generalized adoption of improved sanitation led to significant reductions in the practice of boiling water for drinking. Our analysis suggests that this change is likely a behavioral response to a reduction in the health benefits associated with treating water, which decline and eventually become negligible as local adoption of improved sanitation increases. Estimates of the value of time savings associated with the reduction in water boiling suggest that this adaptation is an additional important benefit of sanitation investments, most of which likely accrues to girls and women.

## 1 Introduction

Access to safe drinking water, sanitation, and hygiene (WASH) in developing countries is recognized as critical for global public health. In 2015, it was estimated that 2.3 billion people lacked access to basic sanitation facilities, with millions of people dying each year due to faecal‐borne diseases (most commonly diarrhea) as a result of inadequate WASH (World Health Organisation/UNICEF 2019).

The WASH research literature to date has largely focused on the effectiveness of various interventions in increasing sanitation coverage and on the associated health improvements (Clasen et al. 2014; Patil et al. 2014; Briceño et al. 2017; Null et al. 2018; Luby et al. 2018; Augsburg and Rodriguez‐Lesmes 2018; Pickering et al. 2019; Cameron et al. 2019, 2021; Cameron et al. 2022), with a smaller, but growing, number of studies examining other outcomes, such as education and cognitive development (Spears and Lamba 2016; Zhang and Xu 2016; Adukia 2017; Coswosk et al. 2019; Orgill‐Meyer and Pattanayak 2020), and labor supply (Wang and Shen 2022). With few exceptions, these studies focus on the impact of own toilet construction on household outcomes.

Improved sanitation however can also generate externalities to surrounding households through a cleaner and healthier local environment. Three recent studies suggest that such externalities matter. Cameron et al. (2022), using data from randomised trials in India, Indonesia, Mali, and Tanzania, find that child height increases once village sanitation coverage exceeds 50%; and Cameron et al. (2021) show, using the same data as this study, that improvements in children's height‐for‐age z‐score (HAZ) mostly reflect the degree of adoption of improved sanitation at local (village) level, rather than their household's decision alone. In a similar vein, Motohashi (2022)'s analysis of a sanitation policy in India that incentivized the construction of over 100 million latrines, finds that its effect in terms of reduced diarrheal mortality is much weaker in areas with poor faecal sludge treatment, because the newly built latrines contribute to river pollution which affects the whole community.

In this paper we contribute to the sparse literature on behavioral adaptations (in terms of changes in preventive health behavior) to the cleaner local environment provided by improved sanitation. Specifically, we present empirical evidence that local sanitation coverage decreases the likelihood that households will boil water before drinking, a behavior adaptation that reflects decreases in the perceived benefits of such behavior, given the persistent high opportunity costs of boiling water.^1^ For example, following widespread latrine construction, people may observe that there is less open defecation and less faecal contamination in their community.

**FIGURE 1:**
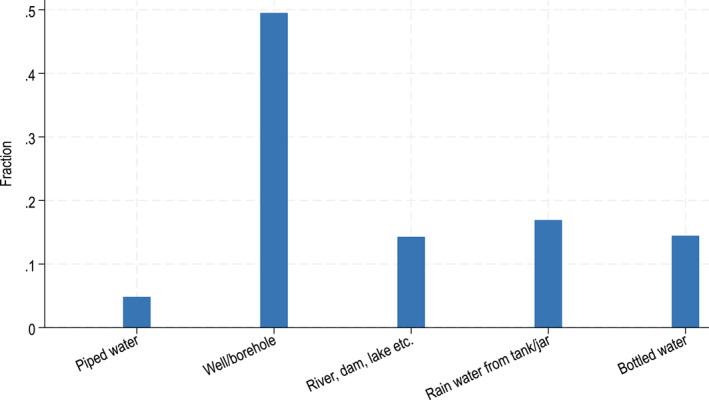
Drinking water sources in the Lao survey sample. The figure plots households' main source of drinking water in the wet season.

We focus on drinking water treatment for several reasons. First, because unsafe drinking water and poor sanitation are equally important contributors to health (World Health Organisation/UNICEF 2019) and microbial contamination of drinking water through contact with faeces poses the greatest risk to drinking water safety. Second, around 80% of the households in our study sample rely on wells or rivers as their source of drinking water. These sources are susceptible to faecal contamination from open defecation and unimproved sanitation facilities. The quality of untreated water from these sources is thus likely to be highly correlated with sanitation coverage and the prevalence of open defecation at village level. Third, more than 60% of households in our sample treated water before drinking at baseline (see Figure 1), with boiling water the most common approach (practiced by 98% of households who treat water). Finally, water boiling also has potentially large implications in terms of time use, given that most households in our sample collect firewood as a primary energy source.

We examine this question in the context of an intervention that combined the randomised allocation of financial incentives for toilet construction with universal provision of Community‐Led Total Sanitation (CLTS) in Lao PDR. CLTS aims to create demand for sanitation through behavior change but, in its standard form, provides no financial assistance for households and communities to construct sanitation infrastructure. That is not the case in our experiment where, on top of CLTS, we randomly encouraged the construction of improved sanitation facilities by providing different financial incentives to either households, communities or both.^2^ Cameron et al. (2021) reports the results of the pre‐registered impact evaluation of the financial incentives on toilet construction and child health. The results we present here were not pre‐registered. The randomised assignment of financial incentives for construction of latrines provides a source of exogenous variation in the adoption of improved sanitation that we exploit to identify the causal effect of improved sanitation coverage on drinking water treatment. The results of our analysis suggest that a ten‐percentage point increase in village sanitation coverage reduces the probability of boiling water for drinking by 3.2% points (approximately 7% of the control mean). We are aware of no other experimental studies that have studied the water treatment decision in conjunction with the adoption of improved sanitation.^3^

Reductions in the practice of water boiling, driven by increased prevalence of improved sanitation at the local level, are potentially concerning, as access to an improved source of drinking water has been shown to improve child health (Jalan and Ravallion 2003) and boiling water is one of the most effective and widely used preventative health measures when water supplies are unsafe (World Health Organisation 2015).^4^ Thus, we examine whether the reduction in water boiling is associated with negative health costs that could offset the positive health impacts of improved sanitation. The association between child health measures and water treatment however appears to be negligible once the majority of households in a village own improved sanitation ‐ as is common in our endline sample.

We then reflect on what benefits may result from this change. A reduction in the time devoted to gathering the firewood needed to boil water is a significant potential benefit. This benefit would largely flow to women and girls as collection of firewood is typically a female task. The act of boiling itself (once firewood has been collected) is also time‐consuming (Clasen et al. 2008). Lighting fires also exposes women and girls to higher risks of respiratory illnesses from indoor pollution (Naeher et al. 2007; Mengersen et al. 2011).

Our estimates of the time saved from reduced firewood collection associated with decreased water boiling suggest that the reduction in water boiling caused by sanitation improvements saves households an average of 54 min daily or 27 h monthly, on this task. This frees women and girls up to spend this time on other activities, such as education or child/elderly care activities. Our conservative estimate of the value of the time savings associated with not boiling water alone are valued at USD 7.30 per household per month (approximately 8% of the median monthly income of households in our sample).

We interpret the observed reduction in water treatment as a rational reaction to improved sanitary conditions and the consequent lower marginal health benefits associated with boiling water, in a context where the costs of this activity remain high. The reductions in water boiling are an additional important benefit of local environmental improvements, generating economic returns and potentially promoting gender equality.

## 2 Community‐Led Total Sanitation (CLTS) With Randomized Financial Incentives

The intervention we exploit combines the randomised allocation of financial incentives for latrine construction with the universal provision of Community‐Led Total Sanitation (CLTS). CLTS aims to bring about the community‐wide elimination of open defecation through inspiring coordinated community action driven by disgust at open defecation and is currently the most widely practiced intervention for improving rural sanitation in developing countries. It has been implemented in nearly 60 countries, 31 of which have incorporated it as a component of national sanitation strategy or policy (Zuin et al. 2019).

In the field, CLTS starts with the “triggering event”—a community meeting in which participants are taken through a carefully facilitated set of activities aimed at helping people understand how faecal contamination spreads from exposed excreta to their living environments (including to food and drinking water), leading to the realisation that people are digesting small amounts of each other's faecal matter with negative health consequences. Households are then encouraged to build hygienic toilets of their own choosing, at their own expense, while communities are encouraged to achieve Open Defecation Free status. This approach emphasizes the creation of demand for sanitation and new social norms that stigmatize open defecation, in contrast to the traditional approach of supplying sanitation hardware, which has been shown to have had limited success (Sah and Negussie 2009). CLTS highlights that faecal‐borne diseases are mainly transmitted through the consumption of untreated water, increasing the likelihood that households' behavior in relation to water will be influenced by local sanitation.^5^

The intervention took place between March 2015 and October 2016 in 160 villages across 10 districts in two provinces (Champasak and Sekong) in rural southern Lao PDR. Financial incentives for latrine construction were randomly assigned to one of four equally sized treatment groups across the 160 villages in our sample: 40 villages per group, stratified by 10 districts, leading to one village per treatment group per district. All villages in both treatment and control groups were universally provided with the CLTS, and the only source of experimental variation is that villages in treatment groups were offered incentives for their latrine construction.

In the first treatment group, villages were assigned to the household incentive scheme. The poorest 30% of households, determined via a score‐card system, were given rebates after verified toilet installation, amounting to roughly 20 USD, or 13% of the price of the lowest‐priced pour‐flush toilet. In the second treatment group, villages were assigned to the village incentive scheme, where a monetary award of between 300 and 500 USD, depending on village population, was offered. This amount was paid to the village administration committee and used at the committee's discretion on any development project, after all households had a hygienic toilet at least 15 m away from their house, along with evidence of regular use. The remaining treated villages were offered both the household‐level rebate and the village‐level award. Villages allocated to the control group received CLTS with no financial incentives. Cameron et al. (2021) analyses this experiment and show that targeted incentives alongside CLTS increased the take‐up of improved sanitation, with significant health spillovers for children.

## 3 Data

We use longitudinal household survey data from 2400 households (15 households per village, and 600 per treatment group) collected in May 2015 and July 2018.^6^ The households were randomly sampled from those in each village with at least one child under 2 years of age at baseline. Each interview took approximately 90 min. A single respondent (most commonly the spouse of the male household head) was asked a variety of questions on household demographics and sanitation. At the end of the interview, caregivers of children in the target age range (0–2 years old at baseline, 3–5 years old at endline) were asked to consent to their child being measured and weighed.

Our outcome of interest is boiling of drinking water at the household level ‐ specifically, a dummy variable which takes the value of one if the respondent boils water before drinking it, and zero otherwise. In our sample, 60% of households treat their drinking water and of these, 98% do so by boiling.

We examine the relationship between better environmental quality (village sanitation coverage) and water boiling. Village latrine coverage is measured using administrative data that is regularly collected by the Provincial Health Department, independently of the research team.

## 4 Empirical Strategy

### 4.1 The Relationship Between Sanitation Coverage and Water Boiling (OLS)

We begin the analysis of the relationship between village sanitation coverage and water boiling by using OLS regression to estimate regressions of the form:

(1) $$\begin{array}{ccc} Boil_{h}^{EL} & = & \alpha +\beta_{1}VillageLatrineCoverage_{v}^{EL}+\beta_{2}X_{h}^{BL}+\beta_{3}V_{v}^{BL}\\ & & +\pi_{1}VillageLatrineCoverage_{v}^{BL}+\pi_{2}ToiletOwnership_{h}^{BL}+\pi_{3}Boil_{h}^{BL}\\ & & +\gamma_{d}+\epsilon_{h} \end{array}$$

where h and v are household and village subscripts, *EL* and *BL* indicate measurement at endline and baseline respectively. Village Latrine Coverage is the percentage of households that own a toilet in each village.^7^ Baseline village latrine coverage and baseline household toilet ownership are included to account for baseline differences in initial latrine ownership which may be correlated with a household's water boiling behavior. We also control for baseline water boiling behavior, $Boil_{h}^{BL}$, and household and village baseline controls, $X_{h}$ and $V_{v}$, respectively. The specific controls included in each specification will be discussed further below. $\gamma_{d}$ is a vector of district fixed effects that accounts for the stratified design. Standard errors are clustered at the village level.

### 4.2 Effect of Sanitation on Water Boiling Behavior (IV Estimates)

The adoption of improved sanitation is likely correlated with a potentially large set of variables, some of which are also plausibly correlated with the decision to boil water before drinking. To address this endogeneity concern, we exploit the randomised assignment to the different incentives as a source of exogenous variation in village sanitation coverage. We use treatment status (three dummy variables indicating randomization into one of the three treatment groups) as instruments for village sanitation coverage. Because the financial incentives were either offered as a direct subsidy for latrine construction or paid to the village administration committee, they are plausibly exogenous and not directly related to the household level decision‐making regarding boiling water. That is, by design, the instruments satisfy the exclusion restriction.

## 5 Results

### 5.1 Summary Statistics, Balance Tests and Attrition

**FIGURE 2:**
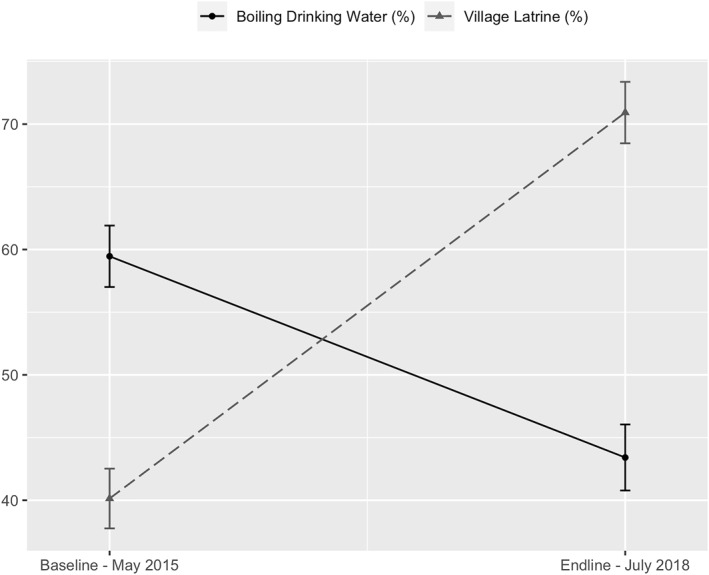
Trends in village latrine coverage and boiling water for drinking. Water boiling is a dummy variable which takes the value of one if the respondent boils water before drinking and zero otherwise. Village latrine coverage is measured by administrative data that is regularly collected by the Provincial Health Department, independently of the research team.

Figure 2 presents a comparison of water boiling and latrine coverage rates at both baseline and endline and clearly illustrates an inverse relationship between the two variables. While the share of households that owned a toilet increased from 45% to 62% between baseline and endline, the share of households that boiled water before drinking decreased from 60% to 45%.

In Tables 1 and 2, we present summary statistics of baseline household and village characteristics, respectively. In the vast majority of pair‐wise comparisons, we cannot reject the null hypothesis of no difference between treatment and control groups. One exception is that households in villages assigned to receive both incentives were less likely to boil water for drinking and more likely to consume bottled water at baseline, compared to households in villages that received no incentives. These differences are however only weakly statistically significant (*p*‐values = 0.097 and 0.077 respectively). Village latrine coverage at baseline is also weakly higher in villages that received the village reward (T2) than in control villages (*p* = 0.10). Other variables that are unbalanced in some comparisons are the household head's gender and ethnicity, the sex ratio within the household, the number of plots of land owned by the household, the use of firewood for cooking, the distance between the household's latrine and drinking water source, and whether the village had a health care program. Conservatively, we control for all unbalanced baseline variables in the statistical analysis.

Sample attrition was 8%, and we find no evidence of differential attrition by treatment status or by whether the household boiled water (our outcome variable) (Table 3).

**TABLE 1:** Balance on baseline household characteristics.

*N* = 2400	C	T1	T2	T3	Differences in means (*p*‐values)					
Intervention	CLTS	CLTS and Household Rebate	CLTS and Village Reward	CLTS and HH Rebate and V Reward	C vs. T1	C vs. T2	C vs. T3	T1 vs. T2	T1 vs. T3	T2 vs. T3
Household size	6.620	6.672	6.597	7.112	0.895	0.944	0.280	0.850	0.383	0.263
	(0.230)	(0.318)	(0.240)	(0.392)						
No. of children	3.313	3.328	3.260	3.570	0.953	0.823	0.401	0.788	0.448	0.313
	(0.168)	(0.190)	(0.170)	(0.256)						
Head male	0.938	0.903	0.943	0.935	0.082*	0.746	0.846	0.059*	0.157	0.649
	(0.010)	(0.017)	(0.012)	(0.014)						
Head age (years)	40.655	41.443	40.612	41.890	0.576	0.970	0.344	0.527	0.758	0.287
	(0.890)	(1.096)	(0.727)	(0.953)						
Head education (category)	1.308	1.292	1.328	1.263	0.867	0.836	0.650	0.728	0.793	0.537
	(0.064)	(0.077)	(0.073)	(0.076)						
Ethnicity: Lao Tai	0.625	0.733	0.553	0.623	0.241	0.457	0.987	0.053*	0.250	0.482
	(0.068)	(0.062)	(0.068)	(0.073)						
Household sex ratio	0.510	0.520	0.514	0.504	0.281	0.703	0.485	0.464	0.067*	0.262
	(0.007)	(0.006)	(0.006)	(0.007)						
Household dependency ratio	0.255	0.253	0.250	0.246	0.798	0.541	0.284	0.725	0.447	0.724
	(0.007)	(0.007)	(0.007)	(0.005)						
Household income (category)	3.738	3.827	3.767	3.680	0.713	0.904	0.822	0.805	0.576	0.738
	(0.168)	(0.175)	(0.170)	(0.196)						
Poor household	0.253	0.223	0.285	0.293	0.601	0.605	0.541	0.292	0.266	0.900
	(0.043)	(0.038)	(0.044)	(0.050)						
Owns house	0.885	0.860	0.875	0.865	0.414	0.734	0.502	0.618	0.869	0.732
	(0.021)	(0.022)	(0.020)	(0.021)						
House floor area (sq m)	54.705	55.235	50.495	54.648	0.873	0.172	0.987	0.122	0.867	0.207
	(2.358)	(2.330)	(1.967)	(2.632)						
Uses firewood for cooking	0.567	0.475	0.682	0.522	0.260	0.134	0.588	0.008***	0.575	0.044**
	(0.057)	(0.058)	(0.051)	(0.060)						
No. plots of land	2.607	2.200	2.608	2.457	0.006***	0.992	0.330	0.012**	0.061*	0.371
	(0.114)	(0.088)	(0.134)	(0.104)						
Owns toilet	0.447	0.442	0.427	0.472	0.934	0.710	0.667	0.807	0.646	0.452
	(0.037)	(0.047)	(0.039)	(0.045)						
Daily open defecation	0.523	0.495	0.532	0.512	0.686	0.901	0.862	0.615	0.820	0.776
	(0.045)	(0.054)	(0.049)	(0.050)						
Dist WC to drinking water (< 10m)	0.078	0.093	0.100	0.130	0.658	0.526	0.179	0.853	0.359	0.455
	(0.023)	(0.025)	(0.026)	(0.031)						
Dist WC to drinking water (> 10m)	0.145	0.205	0.118	0.128	0.103	0.401	0.618	0.019**	0.045**	0.764
	(0.023)	(0.029)	(0.022)	(0.025)						
Boils water	66.833	58.667	57.333	55.000	0.220	0.156	0.097*	0.843	0.608	0.745
	(4.644)	(4.751)	(4.785)	(5.352)						
Other water treatment	0.028	0.027	0.027	0.020	0.932	0.931	0.634	1.000	0.636	0.626
	(0.016)	(0.012)	(0.011)	(0.008)						
No water treatment	0.198	0.245	0.255	0.227	0.425	0.303	0.608	0.871	0.767	0.628
	(0.036)	(0.046)	(0.041)	(0.042)						
Drinks bottled water	0.105	0.142	0.145	0.203	0.459	0.422	0.077*	0.949	0.287	0.315
	(0.033)	(0.037)	(0.037)	(0.044)						

*Note:* Standard errors are clustered at the village level.

* *p* < 0.1.

** *p* < 0.05.

*** *p* < 0.01.

**TABLE 2:** Balance on baseline village characteristics.

*N* = 120	C	T1	T2	T3	Differences in means (*p*‐values)					
Intervention	CLTS	CLTS and Household Rebate	CLTS and Village Reward	CLTS and HH Rebate and V Reward	C vs. T1	C vs. T2	C vs. T3	T1 vs. T2	T1 vs. T3	T2 vs. T3
	Mean/(SE)				Pairwise *t*‐test					
Village sex ratio	0.502	0.510	0.502	0.498	0.422	0.975	0.633	0.565	0.165	0.716
	(0.007)	(0.010)	(0.010)	(0.007)						
% Lao Tai	0.625	0.733	0.553	0.623	0.240	0.461	0.986	0.045**	0.208	0.365
	(0.130)	(0.127)	(0.144)	(0.165)						
Distance to city (kms)	18.700	21.925	25.800	24.875	0.379	0.125	0.271	0.261	0.367	0.807
	(2.511)	(3.700)	(4.297)	(5.051)						
Exit road is dirt	0.650	0.575	0.675	0.625	0.449	0.802	0.829	0.462	0.644	0.658
	(0.102)	(0.081)	(0.119)	(0.135)						
Has rivers	0.625	0.800	0.675	0.700	0.136	0.582	0.538	0.322	0.396	0.816
	(0.142)	(0.081)	(0.148)	(0.135)						
Connected to public piped water	0.275	0.325	0.275	0.275	0.670	1.000	1.000	0.663	0.613	1.000
	(0.086)	(0.086)	(0.117)	(0.083)						
Has centralized water treatment	0.075	0.075	0.100	0.100	1.000	0.596	0.400	0.617	0.396	1.000
	(0.076)	(0.074)	(0.058)	(0.075)						
Had a water program	0.200	0.150	0.125	0.175	0.422	0.428	0.670	0.679	0.524	0.435
	(0.091)	(0.079)	(0.052)	(0.087)						
Latrine coverage	31.163	39.064	47.536	42.816	0.421	0.098*	0.182	0.119	0.554	0.418
	(6.612)	(7.987)	(5.637)	(7.332)						
Open defecation in pond	0.600	0.725	0.625	0.550	0.262	0.800	0.626	0.362	0.125	0.521
	(0.177)	(0.085)	(0.150)	(0.168)						
Had a sanitation materials program	0.200	0.200	0.125	0.100	1.000	0.245	0.308	0.359	0.373	0.654
	(0.109)	(0.101)	(0.058)	(0.074)						
Had a sanitation information program	0.100	0.075	0.150	0.050	0.702	0.606	0.343	0.393	0.578	0.376
	(0.068)	(0.034)	(0.087)	(0.050)						
Had a healthcare program	0.175	0.250	0.175	0.250	0.050**	1.000	0.219	0.351	1.000	0.303
	(0.076)	(0.071)	(0.083)	(0.092)						
Had a deworming program	0.950	0.975	0.950	1.000	0.394	1.000	0.201	0.547	0.324	0.146
	(0.036)	(0.024)	(0.031)	(0.000)						
Observations	40	40	40	40	80	80	80	80	80	80
Number of clusters	10	10	10	10	10	10	10	10	10	10

*Note:* Standard errors clustered at the district level are in brackets below the means.

* *p* < 0.1.

** *p* < 0.05.

***p < 0.01.

**TABLE 3:** Testing for differential attrition.

	Household was re‐interviewed at endline
Treatment group 1	−0.0341
	(0.0311)
Treatment group 2	0.00960
	(0.0258)
Treatment group 3	−0.0301
	(0.0306)
Baseline water boiling (binary)	−0.00181
	(0.0234)
Treatment group 1 * Baseline water boiling	0.0130
	(0.0358)
Treatment group 2 * Baseline water boiling	−0.0264
	(0.0297)
Treatment group 3 * Baseline water boiling	−0.0124
	(0.0344)
Constant	0.963***
	(0.0284)
District fixed effects	Yes
R‐squared	0.02
Mean dependent variable	0.92
Observations	2400

*Note:* Standard errors are clustered at the village level. Dependent variable is whether the household was re‐interviewed at endline.

### 5.2 OLS and IV Estimates of the Relationship Between Village Sanitation Coverage and Water Boiling

The OLS estimates of Equation (1) are presented in Table 4. Column 1 presents the estimates when the only controls included are baseline differences in latrine ownership and water boiling. Column 2 presents the estimates when we additionally include variables that are unbalanced at baseline (discussed above), and in Column 3, we control for all variables listed in Tables 1 and 2.^8^

**TABLE 4:** The relationship between water boiling and village latrine coverage.

	(1)	(2)	(3)	(4)	(5)
	OLS	OLS	OLS	IV	
Village latrine coverage (%, endline)	−0.13**	−0.13**	−0.14***	−0.32*	
	(0.058)	(0.055)	(0.052)	(0.17)	
Water boiling (binary, baseline)	0.29***	0.28***	0.22	0.19	
	(0.040)	(0.039)	(0.48)	(0.49)	
Mean dependent variable	63.6	63.6	63.8	63.8	

Panel B				First stage	Reduced form
Dependent variable				Village latrine coverage (EL)	Household boils drinking water (EL)
Treatment group 1				15.8***	−7.02
				(5.39)	(4.39)
Treatment group 2				15.1**	−4.12
				(5.95)	(4.62)
Treatment group 3				28.3***	−9.10*
				(5.94)	(4.70)
Unbalanced variables at baseline	No	Yes	Yes	Yes	Yes
Full controls	No	No	Yes	Yes	Yes
District fixed effects	Yes	Yes	Yes	Yes	Yes
Kleibergen‐Paap rk LM stat (*p*‐value)				(< 0.01)	
Kleibergen‐Paap rk LM stat (F‐stat)				7.66	
Hansen J‐statistic				0.46	
Hansen J‐statistic *p*‐value				0.79	
Mean dependent variable				68.5	63.8
Observations	1447	1447	1433	1433	1433

*Note:* The unit of observation is a household. The sample is restricted to those households who do not drink bottled water at baseline or endline. Household‐level variables that are unbalanced at baseline include the gender and ethnicity of the household head, the household sex ratio, the use of firewood for cooking, number of plots of land the household owns, distance from drinking water source to latrine, whether the household boils water before drinking and whether the household drinks bottled water. Village‐level variables that are unbalanced at baseline include village latrine coverage and the presence of healthcare programs. Full controls are the full set of variables shown in Tables 1 and 2. All specifications include a control for baseline village latrine coverage. Columns 3 to 5 also include controls for whether the village had a water program or a sanitation materials program in the 3 years prior to endline. The dependent variables in Panel B are: village latrine coverage at endline for the first stage; whether the household boils water for drinking at endline for the reduced form. Standard errors are clustered at the village level.

* *p* < 0.1.

** *p* < 0.05.

*** *p* < 0.01.

The estimates suggest that an improvement in village‐level sanitation coverage is negatively associated with water boiling: a 1% point increase in village latrine coverage is associated with approximately a 0.14% point decrease in the likelihood of water boiling.

Although the point estimates of the coefficients on village sanitation are stable across the different specifications in Columns 1 to 3, these results do not have a causal interpretation. Many village characteristics could drive a relationship between the two variables. For example, more health‐seeking households and communities may be more likely to invest in sanitation and to boil water. The OLS estimates thus may be a biased estimate of the causal effect of sanitation on water boiling behavior. We turn to instrumental variables estimation to identify the causal impacts.

Panel B, Column 4 of Table 4 presents the first stage results and shows that treatment assignment (the different financial incentives) is strongly predictive of increased village sanitation coverage, with the F‐statistic of the Kleibergen‐Paap Wald test above the critical values for weak instruments presented in Stock and Yogo (2005) supporting the relevance of the instruments. We likewise fail to reject the overidentification test of all instruments (Hansen J‐statistic), supporting exogeneity.

Panel A, Column 4 of Table 4 presents the second stage results. A ten‐percentage point increase in village latrine coverage decreases water boiling by about 3.2% points (7% relative to our endline sample mean, *p*‐value = 0.052). While both the OLS and the IV regressions support the conclusion that improved sanitation causes households to reduce water boiling, the IV estimates are larger in magnitude than the corresponding OLS ones. This may reflect that, as discussed above, other things being equal, more health‐seeking households and villages are more likely to invest in both sanitation and boiling water, offsetting the effect of a cleaner environment on boiling of water. The IV approach, by eliminating the endogenous effect of sanitation, removes this offsetting effect, resulting in a larger negative coefficient of sanitation on water boiling.

Column 5 in Table 4 presents the reduced form. It shows negative effects on water boiling associated with treatment. The indicator variable for treatment 3 (CLTS + household rebates + village rewards) is statistically significant at the 10% level (*p* = 0.06) and treatment 1 (CLTS + household rebates) is marginally significant (*p*‐value of 0.11).

### 5.3 Heterogeneity in the Relationship Between Village Sanitation Coverage and Water Boiling

To further explore the relationship between village sanitation coverage and water boiling, we explore how this relationship varies across subsamples that may differ in their perception of the benefits and costs associated with reducing water boiling in response to changes in environmental quality at village level. We consider two behaviors: practice of open defecation at endline and use of firewood as main energy source at baseline.

Table 5 presents the results of estimating the instrumental variables specification over the subsamples defined by the practice of each of these choices.^9^ We interpret these results cautiously as being suggestive of our underlying mechanism, while noting that both open defecation behavior and the use of firewood are potentially endogenous.

**TABLE 5:** Heterogeneity in the relationship between water boiling and village latrine coverage.

	(1)	(2)	(3)	(4)
Estimation	IV	IV	IV	IV
Sample	Open defecation (EL)		Firewood for cooking (BL)	
	No	Yes	Yes	No
Second stage				
Village latrine coverage (%, endline)	−0.49*	−0.22	−0.34*	0.11
	(0.26)	(0.15)	(0.18)	(0.25)
Water boiling (binary, baseline)	0.21	0.24***	0.24***	0.079
	(0.56)	(0.054)	(0.044)	(0.35)
Mean dependent variable	70.67	54.24	67.37	50.33
First stage				
T1	12.8*	21.5***	16.2***	13.9**
	(6.59)	(6.04)	(6.17)	(6.15)
T2	15.1**	15.1**	14.6**	9.57
	(6.44)	(6.93)	(6.70)	(8.34)
T3	23.0***	38.2***	28.2***	29.1***
	(7.17)	(7.04)	(6.70)	(7.27)
Unbalanced variables at baseline	Yes	Yes	Yes	Yes
Full controls	Yes	Yes	Yes	Yes
District fixed effects	Yes	Yes	Yes	Yes
Kleibergen‐Paap rk LM stat (*p*‐value)	0.025	0.0003	0.004	0.006
Kleibergen‐Paap rk LM stat (F‐stat)	3.60	10.6	5.91	5.65
Hansen J‐statistic	0.38	0.82	0.23	6.54
Hansen J‐statistic *p*‐value	0.83	0.66	0.89	0.038
Mean dependent variable	76.99	56.81	69.52	64.79
Observations	832	601	1131	302

*Note:* The unit of observation is a household. The sample is restricted to those households who do not drink bottled water at baseline or endline. Control variables are the same as the full controls in Table 4. The dependent variable in Panel A is whether the household boils their drinking water at endline; in Panel B is village latrine coverage at endline. Standard errors are clustered at the village level.

* *p* < 0.1.

** *p* < 0.05.

*** *p* < 0.01.

First, we estimate the IV specifications over the subsamples of households who engage in open defecation at endline and those who do not. If reduced water boiling is being driven by improved environmental quality, as we suggest, we might expect that those households who still engage in open defecation at endline may be more cautious about reducing water boiling as they would be more acutely aware that the environment is still faecally contaminated. Columns 1 and 2 in Table 5 present results that seemingly support this interpretation. When estimated over the sample of households who do not defecate at endline, the coefficient on village latrine coverage is larger and statistically significant at the 10% level, whereas the estimate for the subsample who still openly defecate at endline is smaller and statistically insignificant.

We then use the same approach to estimate the effect of village latrine coverage, distinguishing between those households who used firewood for cooking (and boling water) at baseline and those who did not. Given the labor costs associated with firewood collection (as discussed in more detail in the next section), we might expect to see greater adaptation to improved environmental quality among those households who rely on that energy source at baseline. The estimates, presented in columns 3 and 4 in Table 5, are supportive of this hypothesis. When estimated over the sample of households who used firewood for cooking at baseline, the coefficient on village latrine coverage is negative and statistically significant at the 10% level, whereas for households who did not use firewood for cooking at baseline the coefficient is small, positive and statistically insignificant.^10^

## 6 Costs and Benefits Associated With Reductions in Water Boiling

Estimates from the previous section suggest that increases in the coverage of sanitation reduce water boiling. In this section, we provide a quantification of the costs and benefits associated with this behavior change in terms of child health status and household time use.

### 6.1 Water Boiling and Child Growth

We examine the association between water boiling and child growth by estimating cross‐sectional OLS regressions on the baseline data in the following form:

(2) $$Health_{c}^{BL}=\alpha +\gamma_{1}Boil_{h}^{BL}+\theta_{1}C_{c}^{BL}+\theta_{2}X_{h}^{BL}+\theta_{3}V_{v}^{BL}+\gamma_{d}+\epsilon_{c}$$

where *Health* indicates the child's height‐for‐age z‐score (HAZ)^11^ for children aged 0–2 years in our sample, and c (h and v) is the child (household and village) subscript. $\theta_{1}$ represents a vector of coefficients on controls for the child, including gender, birth order, and age, as well as whether the child is cared for by his/her parents or by others, caregiver's knowledge about the preventability of diseases and the causes of diarrhea, along with their attitudes towards child and adult open defecation. We also control for the same set of household and village controls as in Tables 1 and 2. $\gamma_{d}$ is a vector of district fixed effects and ϵ is the error term. Standard errors are clustered at village level.

This analysis is necessarily suggestive, and identifies the correlations between the variables. Our estimates in Table 6, columns 1 to 3 suggest that water boiling is associated with better child health, in terms of height‐for‐age z‐score (HAZ), consistent with the literature (e.g., see a recent meta‐analysis by Cohen and Colford (2017)).

**TABLE 6:** OLS estimates of the relationship between water boiling and child growth at baseline.

Dependent variable	(1)	(2)	(3)	(4)
	OLS	OLS	OLS	OLS
	Baseline height for age z‐score			
Water boiling (BL)	0.0436	0.175**	0.170**	0.330**
	(0.0789)	(0.0842)	(0.0854)	(0.129)
Village latrine coverage (BL)				0.0021
				(0.0018)
Village latrine coverage (BL) x Water boiling (BL)				−0.0041*
				(0.0021)
Household toilet ownership (BL)				−0.0439
				(0.135)
Household toilet ownership (BL) x Water boiling (BL)				−0.0126
				(0.148)
Child controls	Yes	Yes	Yes	Yes
Household controls	No	Yes	Yes	Yes
Village controls	No	No	Yes	Yes
District fixed effects	Yes	Yes	Yes	Yes
R‐squared	0.30	0.31	0.31	0.31
Mean dependent variable	−0.63	−0.63	−0.63	−0.63
Observations	2728	2726	2726	2728

*Note:* The unit of observation is a child. Controls for the child include gender, birth order, and age, as well as whether the child is cared for by his/her parents or by others, caregiver's knowledge about the preventability of diseases and the causes of diarrhea, along with their attitudes towards child and adult open defecation. Household and village controls are those listed in Tables 1 and 2. Column 4 only includes household and village controls that had a t‐statistic >1. Standard errors clustered at the village level are in parentheses.

* *p* < 0.1.

** *p* < 0.05.

***p < 0.01.

These results raise the possibility that the positive health effects of the sanitation intervention are attenuated by the behavioral response that households took in reducing water boiling. To further investigate this, we examine how the relationship between water boiling and child health varies with sanitation coverage (column 4). Recall that most households in our sample collect their drinking water from wells and rivers, so we would expect higher sanitation coverage to be associated with higher water quality.

**FIGURE 3:**
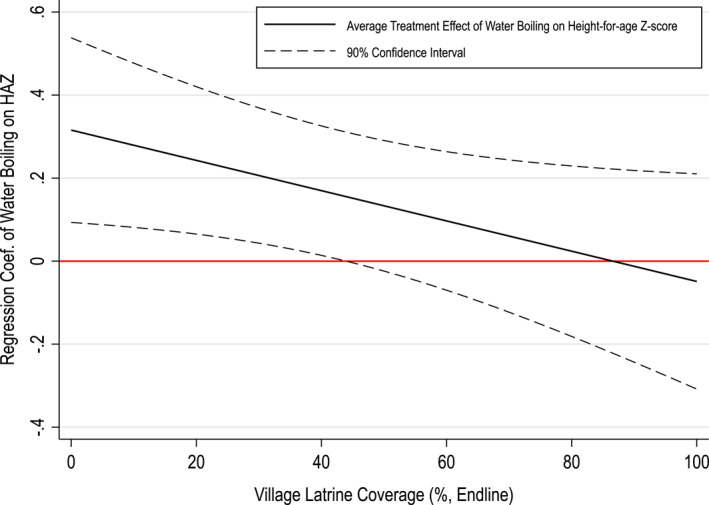
The relationship between water boiling and child growth (height‐for‐age z‐score) as a function of village latrine coverage. This figure graphically illustrates the estimates presented in the final column of Table 6. The black line represents the association between boiling drinking water and child health (height‐for‐age z‐score) across different values of latrine coverage at the village level, while the dashed lines indicate the 90% confidence intervals.

Figure 3 graphically illustrates the estimates presented in the final column in Table 6. It shows that the positive association between water boiling and child height is weaker when village‐level latrine coverage is higher. The association becomes statistically insignificantly different from zero once households live in villages where more than approximately 50% of households own improved sanitation.

**FIGURE 4:**
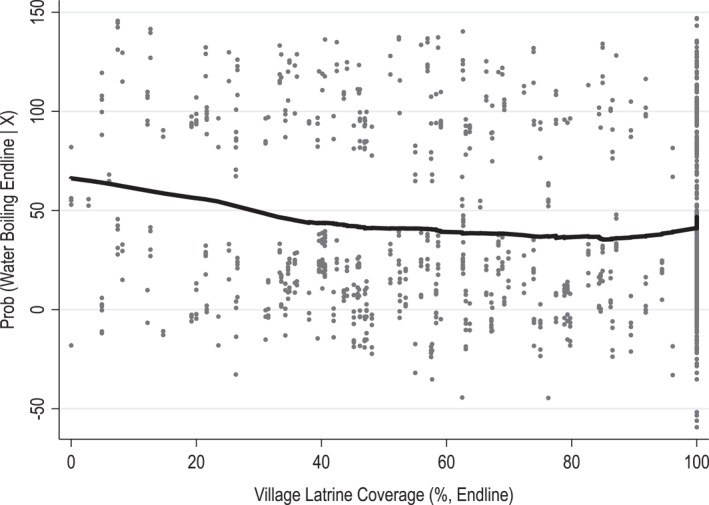
Partial linear regression of water boiling at endline on village latrine coverage at endline. This figure shows a partial linear regression based on Column 2 in Table 4. We use the ‐plreg‐ command introduced by Lokshin (2006). It models endline water boiling as a nonlinear function of village latrine coverage. Each dot represents a household in our sample, while the black curve illustrates the non‐linear trend.

This heterogeneity in the effect of water boiling on child's health is also evident when we split the sample based on the threshold of the village sanitation coverage of 50%. The results are presented in Table 7. They are consistent with those presented in Table 5 and show that the positive association between the child growth measure and household water boiling is only apparent in villages where the sanitation coverage is below 50% (column 1). Once sanitation coverage reaches 50%, the relationship between boiling drinking water and child health becomes statistically insignificant and close to zero in magnitude (column 2). This relationship is mirrored in Figure 4 where there is a clearly nonlinear relationship between village latrine coverage and water boiling: water boiling at endline decreases with latrine coverage until latrine coverage is about 50%.^12^

**TABLE 7:** Sub‐sample anaylsis of the relationship between water boiling and child growth.

	(1)	(2)
	Sample 1	Sample 2
Baseline village latrine coverage	< 50%	> 50%
	OLS	OLS
Dependent variable	Height for age z‐score	
Boiled drinking water at baseline	0.221**	0.0161
	(0.108)	(0.141)
Child controls	Yes	Yes
Household controls	Yes	Yes
Village controls	Yes	Yes
District fixed effects	Yes	Yes
R‐squared	0.33	0.32
Mean dependent variable	−0.54	−0.79
Observations	1716	1010

*Note:* The unit of observation is a child. In our sample, 1010 households (37% of the total) resided in villages with latrine coverage greater than 50% at baseline, while 1925 households (71% of the total) lived in villages with latrine coverage greater than 50% at endline. Standard errors clustered at the village level are in parentheses.

*p < 0.10.

** *p* < 0.05.

***p < 0.01.

The findings above suggest that there exists a threshold of sanitation coverage, estimated to be 50% of sanitation coverage, beyond which health gains from practicing water boiling become negligible. On this basis, we conclude that the reduction in water boiling is unlikely to be associated with significant health costs in our context, given that, at endline, more than 70% of households in our sample live in villages with a cleaner environment where the importance of treating water is negligible (in contrast to 37% of households at the time of the baseline survey). Instead, it seems to be evidence of an informed recalibration, with households changing their behavior in response to improvements in the external environment.

### 6.2 Time Savings From Water Boiling Reduction

Now, we turn to reflect on what benefits may have resulted from this change in behavior. Building on ex ante analysis (World Bank 2013), we focus on the time and energy costs of water boiling, as most households (65%) in our sample collect firewood as a primary energy source for cooking.

To put a value on the benefit of reduced water boiling we use data from the fifth wave of the 2012‐2013 Lao Expenditure and Consumption Survey (LECS5) to calculate the number of hours saved in firewood collection and the foregone income associated with this use of time.^13^ The LECS collects data on the time spent on firewood collection irrespective of its purpose, including cooking, animal food preparation, and heating. Rural households that spend time on firewood collection in LECS5 are similar to those in our own data who report burning firewood for cooking across a range of characteristics, including the demographic structure of the household, household heads' gender, age and education and area of agricultural land farmed (see Appendix Table A4).^14^ Extrapolating from the LECS5 sample to our own, this analysis suggests that household members spend, on average, 3 hours per day on firewood collection.

The hours spent on firewood collection specifically for water boiling is calculated by multiplying this value (3 h) by the share of firewood used for boiling water (14%, based on previous research by Clasen et al. (2008)) and produces a figure of 25 min per day or 13 h per month. In addition, the total time savings should reflect the time taken for the boiling of the water ‐ preparing the fuel, making the fire, and waiting for the water to boil. Drawing on data from (Clasen et al. 2008, 409), users spend on average 2 min preparing the vessel and 5 min starting the fire. In terms of the time the water is boiling, Clasen et al. (2008) finds that the average household that uses wood fuel boils 6.92 L of water per day. The reported boiling time is 6.42 min per liter, and households spend approximately 50% of this time actively tending the fire. This yields an estimated additional 22.21 min per day (6.$92^{*}$6.$42^{*}$0.5). Combining all these figures gives a total of approximately 54 min per day (25 + 29), or 27 h per month, spent collecting firewood, preparing to boil the water and actually boiling the water.

We then multiply this estimated time use by 50% of the Laos national minimum wage to obtain a figure for foregone earnings.^15^ At the time of our field work (2015–2018), the Laos minimum wage for a standard 206‐h work month (48 h per week) was LAK 900,000 (USD 110) (or LAK 4370 per hour). This leads to an estimate of the opportunity cost of collecting firewood for boiling water of LAK 59,000 (USD 7.30) per household per month, which is equivalent to approximately 8% of the median value of household monthly income in our sample (LAK 750,000).

As in other contexts, there is a clear gender division in who is responsible for firewood collection, with women and girls participating in this activity in 73% households that collect firewood and being solely responsible for firewood collection in 60% of these households (Table A6). Table 8 presents some evidence of how individuals might have otherwise allocated their time, had they not had to collect firewood. Time spent on firewood collection is negatively associated with time spent on education (column 1), and this negative association is almost one‐to‐one for individuals younger than 18 (column 2). Individuals who are older than 18 tend to spend their time on children/elderly care activities (column 4). The opportunity cost of firewood collection is maybe then best thought of in terms of educational opportunities and time spent in caring activities, rather than in terms of foregone earnings.

**TABLE 8:** Opportunity costs of collecting firewood.

Dependent variable	(1)	(2)	(3)	(4)
	Time spent on education		Time spent caring for dependent family members	
Time spent on firewood collection	−0.310***	−0.112***	−0.176***	−0.196***
	(0.0291)	(0.0241)	(0.034)	(0.040)
Age below 18 (binary)		3.102***		−0.223***
		(0.168)		(0.093)
Time spent on firewood collection x Age below 18		−0.771***		0.087
		(0.0861)		(0.058)
Individual controls	Yes	Yes	Yes	Yes
Household fixed effects	Yes	Yes	Yes	Yes
R‐squared	0.26	0.41	0.09	0.09
Mean dependent variable (hours per day)	1.1	1.1	1.85	1.85
Observations	2946	2946	2946	2946

*Note:* The unit of observation is an individual. The “dependent family members” in the last two columns include both children and the elderly. Individual controls include gender and the relationship to the household head (head, spouse, parents, children, siblings, other relatives, not relative). Columns (1) and (4) also include a continuous control for the age of the individual. Standard errors clustered at the household level are in parentheses.

* *p* < 0.10.

**p < 0.05.

*** *p* < 0.01.

### 6.3 Other Benefits of Reduced Water Boiling

Reductions in water boiling also potentially generate other societal benefits, including reductions in the depletion of forests when, as in our case, firewood is the main source of energy used by the household. Rural families in Laos consume approximately 183 kg of firewood per month for cooking, with 25.6 kg used for boiling drinking water (World Bank 2013)). This leads to an annual consumption of 0.3 million tons of firewood (0.65–1.3 cubic meters per household year). Reductions in boiling drinking water, in this context means that households are reducing the release of large amounts of carbon into the atmosphere.

Reductions in the burning of firewood may also generate additional health benefits as the use of this source of energy leads to high concentrations of particulate matter (PM10) and nitrogen dioxide (${\text{NO}}_{2}$) which are about 25 times higher than the World Health Organization's (WHO) 24‐h mean guidelines. The almost 1 hour per day women and girls spend in the household cooking area boiling water exposes them to a higher risk of associated health hazards. Mengersen et al. (2011) find that a wide range of symptoms of respiratory illness in women and children aged 1–4 years in Lao PDR are positively associated with exposure to indoor cooking. Table A7 shows that, in our survey data, having an indoor kitchen and firewood as the main energy source for cooking is associated with a 6% point (40%) increase in the probability that children under 5 in the household had a cough in the previous 7 days.^16^

## 7 Discussion

This paper contributes to the sparse literature on behavioral adaptations to a cleaner local environment. We find that improvements in village sanitation coverage, generated by the randomised allocation of financial incentives for latrine construction alongside universal provision of Community‐Led Total Sanitation (CLTS), led to a significant reduction in boiling water for drinking.

In relation to the costs and benefits associated with reductions in water boiling, our analysis suggests that the link between child health and water treatment becomes negligible when most village households have improved sanitation, a condition prevalent in our endline sample. On that basis the reduction in water boiling is unlikely to be associated with substantial health costs in our context. Rather, the reduction seems to reflect a rational behavioral response to a cleaner local environment. This change in behavior is an additional benefit of sanitation investments, generating time savings that are disproportionately enjoyed by women and girls in our context.

Our study has powerful implications for cost‐benefit analysis of sanitation investments. Governments in developing countries continue to devote resources to increasing hygienic toilet use. The existing literature (e.g., Dickinson et al. (2015)) has shown that latrines are highly cost‐effective investments, mainly based on returns to child health, education, and adult time‐savings. The findings in this paper suggest that previous studies may have understated the benefits of improved sanitation by not accounting for additional behavioral change flowing from the reduced need for boiled water. In addition to the time‐savings of households, reduced burning of firewood for water boiling is likely to generate significant environmental and health benefits. Finally, while gender inequality has deep social roots, our study reveals that improved sanitation has the potential to contribute to gender parity in rural areas by freeing women and girls up from the time‐consuming demands of firewood collection.

## Conflicts of Interest

The authors declare no conflicts of interest.

## Data Availability

The data that support the findings of this study are available from Lisa Cameron (lisa.cameron@unimelb.edu.au) or An Huang (anhuang96@gmail.com) upon reasonable request.

## Endnotes

^1^
The demand for preventive health behaviors has been shown to be highly sensitive to costs (Ashraf et al. 2010; Kremer et al. 2011; Berry et al. 2020) and so may be similarly sensitive to reductions in the perceived benefits.

^2^
Ethics approval for the study was received from the Lao National Institute of Public Health's National Ethics Committee for Health Research (NECHR), No. 39/2014 and No. 85/2018.

^3^
The closest study to ours is Bennett (2012) which studied the substitutability between clean water and household sanitary behavior in the Philippines in a non‐experimental setting, finding that the provision of piped water reduced household investment in sanitation.

^4^
The child health benefits associated with improved water supply have also been shown to be associated with higher parental investments in education (Gibson and Lawson 2011).

^5^
That CLTS highlights the relationship between sanitation and drinking water does not pose a problem for the evaluation as all households—including households in the control group—receive CLTS and its health messaging. The availability of the financial incentives accompanying CLTS is randomised and generates the exogenous variation in sanitation coverage which we exploit in the analysis. The construction of sanitation facilities could directly improve drinking water provision if it is accompanied by other infrastructure developments that make it easier for people to get access to clean water, for example, road access or piping of water. This is, however, unlikely in our context as the type of toilet thatwas almost uniformly built drained to households' private tanks. The funding that accompanied CLTS in treatment villages was insufficient to cover the cost of the construction of any public infrastructure and we are not aware of any such infrastructure being built. Further, our data shows no evidence of large increases in piped drinking water.

^6^
The intervention was only implemented in two villages prior to May 2015 and was only at its very early stages in those villages.

^7^
We focus on village sanitation coverage, rather than household toilet ownership, as the prevalence of sanitation in a community is what matters most for local environmental quality (Augsburg and Rodriguez‐Lesmes (2018); Bakhtiar et al. (2021); Cameron et al. (2021); Cameron et al. (2021); Cameron et al. (2022); Pakhtigian et al. (2022)).

^8^
We also control for whether the village had a water program or sanitation materials program in the 3 years prior to endline as this could affect both sanitation and water treatment behavior. The estimation sample is restricted to households who do not consume bottled water at baseline and endline as the decision to drink bottled water in low and middle income countries is often strongly associated with income, social norms and status and so distinct from the health‐seeking decision to boil water (Tabar et al. 2023). The consumption of bottled water increased between baseline and endline and, as Table A1 shows, was not affected by treatment status. As a sensitivity analysis, Table A2 in the appendix presents results where we include those who drink bottled water at endline (but not those who drink bottled water at baseline as they do not face the decision as to whether to boil their drinking water). The results are very similar to those in Table 4 but are less precisely estimated. Table A3 presents results estimated over the entire sample, including those who boil water at baseline or endline, and treating both boiling water and drinking bottled water as forms of water treatment. Again the point estimate on village latrine coverage is very similar to in Table 4 and, reflecting the larger sample size, now statistically significant at the 5% level.

^9^
We thank an anonymous referee for suggesting the specifications presented in this section.

^10^
Note that the diagnostic tests are less strongly supportive of the validity of the instruments over the sub‐samples. Particularly, the Hansen J‐test for the subsample who do not use firewood for cooking suggests that the instruments may not be valid.

^11^
We used the ‐zscore06‐ command in Stata (Leroy 2011) to estimate height‐for‐age z‐scores.

^12^
We cannot conduct the instrumental variables estimation with boiling drinking water as the dependent variable (analogous to Table 4) separately for observations in villages with sanitation coverage above and below 50% at endline, as the instruments lose predictive power in the sub‐samples.

^13^
As our survey data do not include information on time use.

^14^
An analysis of the determinants of the number of hours spent on firewood collection finds households with a male head, education of primary school level or lower, and a greater number of adult women in the household spend more hours on this task. Time spent on firewood collection seem to (weakly) decrease with education. See Appendix Table A5.

^15^
Whittington and Cook (2019) recommend applying 50% of the after‐tax realised wage, rather than 50% of the minimum wage, to better reflect the opportunity cost of time in low income countries. However, our dataset does not include information on time use, such as hours worked per month, which prevents us from calculating realised hourly wages. Nonetheless, the national minimum wage in Laos (LAK 900,000 per month) is lower than average earnings of rural female workers in casual agricultural employment in Laos (average monthly income of LAK 1,421,000, according to the (Lao Statistics Bureau 2018, 61)), suggesting that our estimate is relatively conservative.

^16^
We do not have data on adult respiratory illness.

## Appendix A

**TABLE A1:** Treatment does not affect bottled water consumption.

Dependent variable	(1)
	OLS
	Drinks bottled water (EL)
Treatment group 1	0.0072
	(0.045)
Treatment group 2	−0.024
	(0.036)
Treatment group 3	−0.020
	(0.041)
Unbalanced variables at baseline	Yes
Full controls	Yes
District fixed effects	Yes
Mean dependent variable	0.297
Observations	2195

*Note:* The unit of observation is a household. Control variables are the same as in Table 4. Standard errors are clustered at the village level.

* *p* < 0.1.

** *p* < 0.05.

*** *p* < 0.01.

**TABLE A2:** The relationship between village latrine coverage and water boiling—Sensitivity to sample selection.

Panel A	(1)	(2)	(3)	(4)	(5)
	OLS	OLS	OLS	IV	
	Dependent variable: Household boils water (EL)				
Village latrine coverage (%, endline)	−0.14**	−0.12**	−0.10*	−0.28	
	(0.062)	(0.061)	(0.058)	(0.18)	
Water boiling (binary, baseline)	0.22***	0.22***	0.27***	0.24***	
	(0.035)	(0.036)	(0.074)	(0.079)	
Boiling or bottled water (binary, baseline)					
Mean dependent variable	50.0	50.0	50.1	50.1	
Observations	1893	1893	1878	1878	

**Panel B**				**First stage**	**Reduced form**
**Dependent variable**				**Village latrine coverage (EL)**	**Household boils drinking water (EL)**
Treatment group 1				14.7***	−6.75*
				(4.76)	(4.06)
Treatment group 2				13.6**	−2.56
				(5.24)	(4.45)
Treatment group 3				27.0***	−7.36
				(5.31)	(4.74)
Unbalanced variables at baseline	No	Yes	Yes	Yes	Yes
Full controls	No	No	Yes	Yes	Yes
District fixed effects	Yes	Yes	Yes	Yes	Yes
Kleibergen‐Paap rk LM stat (*p*‐value)	—	—	—	(< 0.01)	—
Kleibergen‐Paap rk LM stat (F‐stat)	—	—	—	8.75	—
Hansen J‐statistic	—	—	—	0.93	—
Hansen J‐statistic *p*‐value	—	—	—	0.63	—
Mean dependent variable				68.3	50.1
Observations				1878	1878

*Note:* The unit of observation is a household. All specifications are estimated over the sample restricted to those households who do not drink bottled water at baseline. Control variables are the same as in Table 4. Standard errors are clustered at the village level.

* *p* < 0.1.

** *p* < 0.05.

*** *p* < 0.01.

**TABLE A3:** The relationship between village latrine coverage and water boiling/bottled water.

	(1)	(2)	(3)	(4)	(5)
Panel A	OLS	OLS	OLS	IV	
Dependent variable	Household boils water or drinks bottled water (EL)				
Village latrine coverage (%, endline)	−0.077	−0.093*	−0.13**	−0.30**	
	(0.053)	(0.053)	(0.051)	(0.15)	
Boil of bottled water (baseline)	0.26***	0.30***	0.22***	0.22***	
	(0.031)	(0.034)	(0.029)	(0.029)	
R‐squared	0.20	0.22	0.26	0.26	
Mean dependent variable	74.85	74.85	75.08	75.08	
Observations	2211	2211	2195	2195	
**Panel B**				**First stage**	**Reduced form**
**Dependent variable**				**Village latrine coverage (EL)**	**Household boils water or drinks bottled water (EL)**
Treatment group 1				14.1***	−4.89
				(4.62)	(3.99)
Treatment group 2				13.6***	−2.96
				(4.94)	(3.62)
Treatment group 3				27.2***	−8.07**
				(4.78)	(4.01)
Unbalanced variables at baseline	No	Yes	Yes	Yes	Yes
Full controls	No	No	Yes	Yes	Yes
District fixed effects	Yes	Yes	Yes	Yes	Yes
Kleibergen‐Paap rk LM stat (*p*‐value)				(<0.01)	
Kleibergen‐Paap rk LM stat (F‐stat)				10.95	
Hansen J‐statistic				0.25	
Hansen J‐statistic *p*‐value				0.88	
Mean dependent variable				70.9	75.1
Observations				2195	2195

*Note:* The unit of observation is a household. The results presented are estimated over the entire sample. Control variables are the same as in Table 4. Standard errors are clustered at the village level.

* *p* < 0.1.

** *p* < 0.05.

*** *p* < 0.01.

**TABLE A4:** Comparison of LECS data and survey data.

	Lao expenditure and consumption survey (LECS)		Survey sample	
	Mean/SD	Observations	Mean/SD	Observations
Time spent on firewood collection (hours)	2.97	633		
	(3.28)			
Firewood only collected by women and girls (%)	61.77	633		
	(48.63)			
Women and girls participated in firewood collection (%)	73.62	633		
	(44.11)			
Household size (count)	6.21	633	7.02	1567
	(2.55)		(3.58)	
Head male	0.942	633	0.946	1567
	(0.235)		(0.227)	
Number of female household members	3.12	633	3.54	1567
	(1.60)		(2.06)	
Head age (years)	45.87	633	39.49	2400
	(12.44)		(13.20)	
Head's education: Less than primary school	0.246	622	0.189	1567
	(0.431)		(0.008)	
Head's education: Primary school	0.545	622	0.519	1567
	(0.498)		(0.500)	
Head's education: Lower secondary school	0.162	622	0.153	1567
	(0.369)		(0.361)	
Head's education: Upper secondary school	0.024	622	0.078	1567
	(0.154)		(0.268)	
Head's education: Tertiary and vocational	0.023	622	0.031	1567
	(0.148)		(0.176)	
Floor area of house (sq meters)	56.11	633	47.55	2400
	(59.81)		(26.37)	
Agricultural land area	2.20	633	1.84	1567
	(2.45)		(3.13)	

*Note:* The unit of observation is a household. The sample for Lao expenditure and consumption survey is rural households who spent time on firewood collection in the last 24 h (at the time of survey administration). The sample for the survey data is households who report using firewood for cooking. Standard deviations are in parentheses.

**TABLE A5:** Correlates of household firewood collection.

	(1)	(2)	(3)	(4)
Dependent variable: Hours household spends on firewood collection				
Household head characteristics				
Male	1.068	1.350*	1.072***	1.047***
	(0.659)	(0.759)	(0.255)	(0.227)
Age (years)	0.00331			
	(0.0117)			
Married	−0.567	−0.318		
	(0.573)	(0.771)		
Literate	0.0663			
	(0.410)			
Highest education ‐ primary school	−0.184	−0.128	−0.126	
	(0.415)	(0.273)	(0.275)	
Highest education ‐ lower secondary	−0.651	−0.947**	−0.956**	
	(0.562)	(0.413)	(0.408)	
Highest education ‐ upper secondary and above	−0.232	−0.603	−0.595	
	(0.602)	(0.500)	(0.504)	
Highest education ‐ lower secondary and above				−0.775**
				(0.339)
Ethnicity ‐ Lao Tai	−0.160			
	(0.304)			
No. of household members	0.104			
	(0.105)			
No. of adult female household members	0.265	0.416**	0.412**	0.417**
	(0.207)	(0.159)	(0.156)	(0.153)
No. of female household members under 18 years	0.105			
	(0.131)			
Household owns house	0.118			
	(0.565)			
House floor area (sqm)	0.00245			
	(0.00384)			
House has an indoor kitchen	0.101			
	(0.257)			
Agricultural land area	0.0776			
	(0.104)			
Runs a non‐farm business	0.0667			
	(0.400)			
Constant	0.228	0.700	0.674	0.550
	(1.054)	(0.403)	(0.422)	(0.372)
N	672	686	686	686

*Note:* We present results from ordinary least squares regresion. All specifications include provincial fixed effects. Column 1 reports results with the full set of controls shown. Column 2 includes only those independent variables which had a t‐statistic > 1 in column 1. Column 3 includes only those independent variables which had a t‐statistic > 1 in column 2. A test of equality of the coefficients on having a household head with a lower secondary education and having a head with at least upper secondary education failed to reject that they are equal. Column 4 imposes equality of the aforementioned educational category variables. Standard errors are clustered at the province level and are shown in parentheses.

* *p* < 0.10.

** *p* < 0.05.

*** *p* < 0.01.

**TABLE A6:** Who collects firewood?

	%
Type 1: Households that have adults only (*N* = 181)	
Women are solely responsible for firewood collection	60.4
Women participate in firewood collection	72.5
Type 2: Households that have children (<=18 years old), but boys only (*N* = 139)	
Women are solely responsible for firewood collection	45.5
Women participate in firewood collection	59.7
Type 3: Households that have children (<=18 years old), but girls only (*N* = 149)	
Women and girls are solely responsible for firewood collection	69.7
Women and girls participate in firewood collection	79.6
Type 4: Households that have children (<=18 years old), with both boys and girls (*N* = 164)	
Women and girls are solely responsible for firewood collection	70.7
Women and girls participate in firewood collection	82.1

*Note:* The unit of observation is a household. The sample is from Lao expenditure and consumption survey where rural households who spent time on firewood collection in the last 24 h (at the time of survey administration). This table suggests that women and girls are mainly responsible for firewood collection, and hence, they bear most of the associated costs.

**TABLE A7:** Associations between using firewood for cooking and children's respiratory illness.

	(1)	(2)	(3)
Dependent variable: Child aged under 5 years had a cough in the 7 days prior to the survey			
	Marginal effects		
Firewood is the main energy source for cooking and have an indoor kitchen	0.0644*	0.0656**	0.0639**
	(0.0332)	(0.0325)	(0.0324)
Child controls	Yes	Yes	Yes
Household controls	No	Yes	Yes
Village controls	No	No	Yes
District fixed effects	Yes	Yes	Yes
Mean dependent variable	0.16	0.16	0.16
Observations	2728	2726	2726

*Note:* The unit of observation is a child. We report marginal effects from probit estimation. Child, household and village control variables are the same as in Table 6. All specifications also include controls for firewood being the main energy source for cooking and whether the household has an indoor kitchen. Standard errors clustered at the village level and shown in parentheses.

* *p* < 0.10.

** *p* < 0.05.

*** *p* < 0.01.
